# OLDER ADULTS’ SOCIAL RELATIONSHIPS AND WELL-BEING IN A DIGITAL WORLD

**DOI:** 10.1093/geroni/igz038.050

**Published:** 2019-11-08

**Authors:** Shannon T Mejia, Sara J Czaja

**Affiliations:** 1 University of Illinois, Urbana-Champaign, Champaign, Illinois, United States; 2 Weill Cornell Medicine, New York, New York, United States

## Abstract

As adults age into a digitally connected world, communication technologies such as the internet, email, social media, and video chats offer new opportunities to connect with others. The implications of older adults’ use of technology in the context of their social relationships—such as the implications for social integration, the relational circumstances of technology adoption, implications for daily experiences of well-being, and opportunities to form new relationships—are less understood. This symposium brings together diverse and complementary perspectives on the contribution of technology to older adults’ social experiences. We begin with inquiry into implications of internet use for social integration. Hees and colleagues use data from the German Ageing Survey to examine how internet use is associated with change in loneliness over a three-year period in older adults who are either before or after retirement. Our symposium continues with papers on technology use within the context of older adults’ existing close relationships. Chopik examines individual and dyadic predictors of technology adoption. Mejía and colleagues consider the implications for digital social interactions for older adult’s well-being on that day. Our final paper discusses the potential for technology to aid in the development of new relationships. Rogers and colleagues describe findings from their OneClick.chat project, a web-based video chat application that connects older adults based on their shared interests. Our session concludes with a discussion led by Czaja, who will integrate the four papers and discuss the challenges and opportunities of using technology to support older adults’ social relationships and well-being.

